# Tetraspanin CD82 Correlates with and May Regulate S100A7 Expression in Oral Cancer

**DOI:** 10.3390/ijms25052659

**Published:** 2024-02-24

**Authors:** Kiran Kumar Reddi, Weiqiang Zhang, Shokoufeh Shahrabi-Farahani, Kenneth Mark Anderson, Mingyue Liu, David Kakhniashvili, Xusheng Wang, Yanhui H. Zhang

**Affiliations:** 1 Department of Bioscience Research, College of Dentistry, University of Tennessee Health Science Center, 875 Union Ave, Memphis, TN 38163, USA; 2 Department of Pediatrics, College of Medicine, University of Tennessee Health Science Center, Memphis, TN 38163, USA; 3 Department of Physiology, College of Medicine, University of Tennessee Health Science Center, Memphis, TN 38163, USA; 4 USDA-ARS, Pollinator Health in Southern Crop Ecosystem Research Unit, 141 Experiment Station Road, P.O. Box 346, Stoneville, MS 38776, USA; 5 Department of Diagnostic Sciences, College of Dentistry, University of Tennessee Health Science Center, 875 Union Ave, Memphis, TN 38163, USA; 6 The Proteomics & Metabolomics Core Facility, University of Tennessee Health Science Center, 71 S. Manassas, Suite 110, Memphis, TN 38163, USA; 7 Department of Genetics, Genomics & Informatics, University of Tennessee Health Science Center, 71 S. Manassas, Room 410H, Memphis, TN 38163, USA

**Keywords:** oral squamous cell carcinoma, protein expression, gene expression, transcriptomics, oral pathology, proteomics, CRISPR-Cas9, knockout, KAI1, epithelial

## Abstract

Many metastatic cancers with poor prognoses correlate to downregulated CD82, but exceptions exist. Understanding the context of this correlation is essential to CD82 as a prognostic biomarker and therapeutic target. Oral squamous cell carcinoma (OSCC) constitutes over 90% of oral cancer. We aimed to uncover the function and mechanism of CD82 in OSCC. We investigated CD82 in human OSCC cell lines, tissues, and healthy controls using the CRISPR-Cas9 gene knockout, transcriptomics, proteomics, etc. CD82 expression is elevated in CAL 27 cells. Knockout CD82 altered over 300 genes and proteins and inhibited cell migration. Furthermore, CD82 expression correlates with S100 proteins in CAL 27, CD82KO, SCC-25, and S-G cells and some OSCC tissues. The 37–50 kDa CD82 protein in CAL 27 cells is upregulated, glycosylated, and truncated. CD82 correlates with S100 proteins and may regulate their expression and cell migration. The truncated CD82 explains the invasive metastasis and poor outcome of the CAL 27 donor. OSCC with upregulated truncated CD82 and S100A7 may represent a distinct subtype with a poor prognosis. Differing alternatives from wild-type CD82 may elucidate the contradictory functions and pave the way for CD82 as a prognostic biomarker and therapeutic target.

## 1. Introduction

Tetraspanin CD82 is a metastasis suppressor. Its expression is often downregulated in advanced stages of many cancers, such as lung, pancreatic, breast, prostate, colon, and ovarian cancers, and this loss of expression is associated with a poor prognosis; however, in leukemia and some advanced solid tumors, it is upregulated [1,2,3,4,5]. According to a review of 64 studies, 83% of them reported that CD82 expression was downregulated in cancer tissues. A total of 16% of studies showed that CD82 expression varied during cancer progression [3]. Understanding the expression regulation and functions of CD82 in metastatic cancer is essential to target CD82 as a prognostic biomarker or therapeutic.

CD82 is poorly understood in oral cancer, and there are limited studies on its role in oral squamous cell carcinoma (OSCC). Patients with oral cancer suffer from a low survival rate and poor quality of life. The 5-year relative survival rate is only 68% [6]. OSCC accounts for over 90% of all oral cancer cases. CD82 expression is downregulated or negative in most OSCC tissues, and it is upregulated in some tissues [7,8,9,10,11,12,13].

We aimed to uncover the function and mechanism of CD82 in OSCC, particularly in CD82-upregulated metastatic OSCC with a poor prognosis. We used two OSCC cell lines, namely CAL 27 and SCC-25; one healthy human gingival epithelial cell line—Smulow-Glickman (S-G)—as the control; and seven Formalin-Fixed Paraffin-Embedded (FFPE) human OSCC tissues. Both CAL 27 and SCC-25 cells are human papillomavirus (HPV) negative (poorer prognosis than HPV positive) with mutated TP53 and CDKN2A genes [14,15,16,17,18]. The methods we used include CRISPR-Cas9 gene knockout, transcriptomics, and proteomics.

## 2. Results

### 2.1. CD82 Is Upregulated in CAL 27 Cells

**Figure 1:**
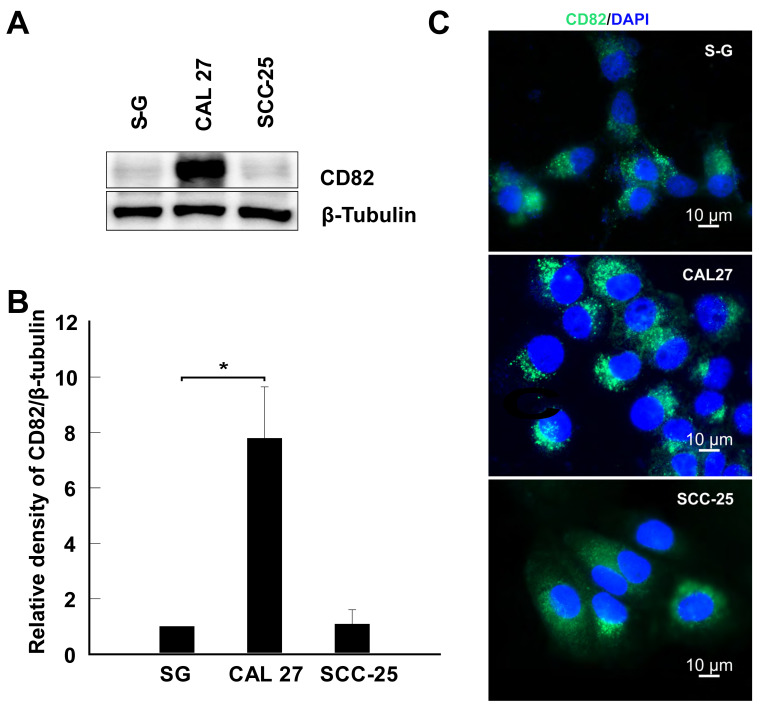
Tetraspanin CD82 expression varies in OSCC cells and is elevated in CAL 27 cells. (**A**) A representative blot showing the expression of CD82 in S-G, CAL 27, and SCC-25 cells. (**B**) Quantification of CD82 expression blots using ImageJ. CD82 expression levels were quantified relative to the levels in S-G cells. *: *p* < 0.05; *n* = 3. (**C**) Representative immunofluorescent images of CD82 expression in S-G, CAL 27, and SCC-25 cells. TS82b was used as the primary antibody in these experiments. HRP-conjugated and FITC-conjugated anti-mouse IgGs were the secondary antibodies used in Western blots and immunostaining.

We tested CD82 protein expression in two human OSCC cell lines, CAL 27 and SCC-25, and S-G control cells. Western blotting and immunofluorescence imaging data showed that the CD82 protein expression levels vary in these three cell lines; it was upregulated (7.8-fold) in CAL 27 cells and similar in SCC-25 cells compared to S-G cells (Figure 1A–C).

### 2.2. Generating CD82 Knockout CAL 27 Cells Using CRISPR-Cas9

**Figure 2:**
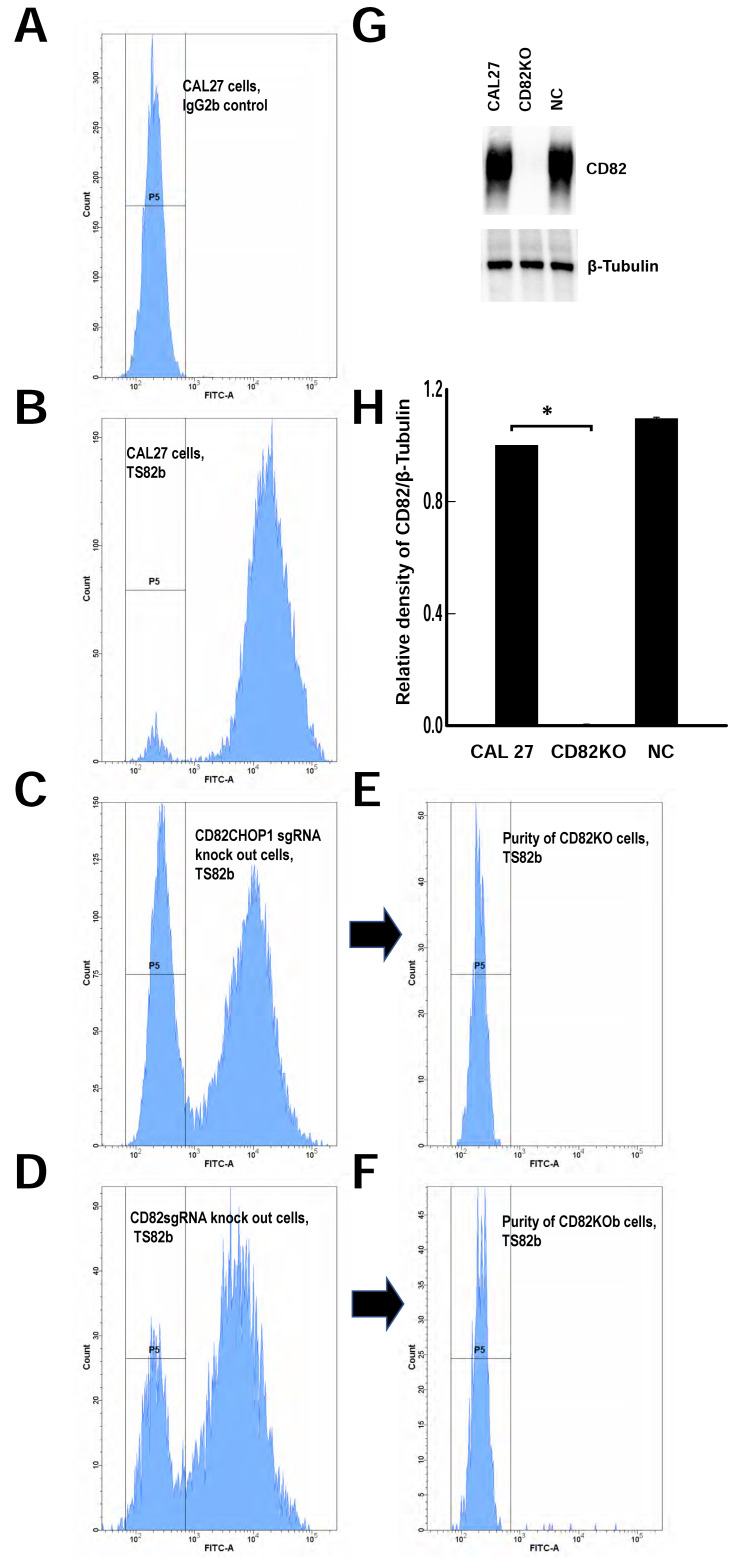
CAL 27 CD82KO and CD82KOb cells were established using a CRISPR-Cas9 kit with TrueGuide sgRNAs. FACS data of (**A**) unstained CAL 27 cells (IgG2b primary antibody, FITC-conjugated anti-mouse IgG secondary antibody). (**B**) CAL 27 cells stained with TS82b primary antibody and FITC-conjugated anti-mouse IgG secondary antibody. (**C**) CD82KO cells stained with TS82b primary antibody and FITC-conjugated anti-mouse IgG secondary antibody. A total of 37.6% of the CD82CHOP1 sgRNA knockout CAL 27 cells shifted to the CD82 negative cell population. (**D**) CD82KOb cells stained with TS82b primary antibody and FITC-conjugated anti-mouse IgG secondary antibody. A total of 26.3% of the CD82sgRNA knockout CAL 27 cells shifted to the CD82 negative cell population. (**E**) Purity of CD82KO cells. CD82KO cells were enriched by collecting the top 5% of the negative population of the CD82CHOP1 sgRNA knockout CAL 27 cells using FACS. (**F**) Purity of CD82KOb cells. CD82KOb cells were enriched by collecting the top 3% of the CD82 negative population of the CD82sgRNA knockout CAL 27 cells using FACS. (**G**) A representative blot showing CD82 expression in CAL 27, CD82KO, and negative control (NC) cells. (**H**) Quantification of CD82 expression from blots using ImageJ. CD82 expression levels were quantified relative to the level in CAL 27 cells. *: *p* < 0.05; *n* ≥ 3 for Western blots. CD82KO cells: CAL 27 cells transfected with the CRISPR-Cas9 CD82CHOP1 sgRNA; CD82KOb cells: CAL 27 cells transfected with the CRISPR-Cas9 CD82 sgRNA; negative control cells (NC): CAL 27 cells transfected with the CRISPR-Cas9 negative control sgRNA.

To investigate the roles of CD82 in CD82-upregulated OSCC, we knocked out the CD82 gene in CAL 27 cells using CRISPR-Cas9. A flow cytometry analysis demonstrated the successful knockout of CD82 from CAL 27 cells using CD82CHOP1 sgRNA or CD82sgRNA, with higher efficiency observed for CD82CHOP1 sgRNA. A total of 37.6% of the CD82CHOP1 sgRNA knockout CAL 27 cells shifted to a CD82 negative cell population compared to a 26.3% shift using CD82sgRNA (Figure 2A–D). Since CD82CHOP1 sgRNA targets isoform 1 (NM_002231.3) of CD82, we speculate that isoform 1 is the major isoform of CD82 in CAL 27 cells.

CD82KO and CD82KOb cell lines were established by collecting the CD82-negative populations from CD82CHOP1 sgRNA knockout and CD82 sgRNA knockout CAL 27 cells through FACS. (Figure 2E,F). Immunoblotting confirmed the absence of CD82 in CD82KO cells (Figure 2G,H).

### 2.3. CD82 Knockout Inhibits Cell Migration

**Figure 3:**
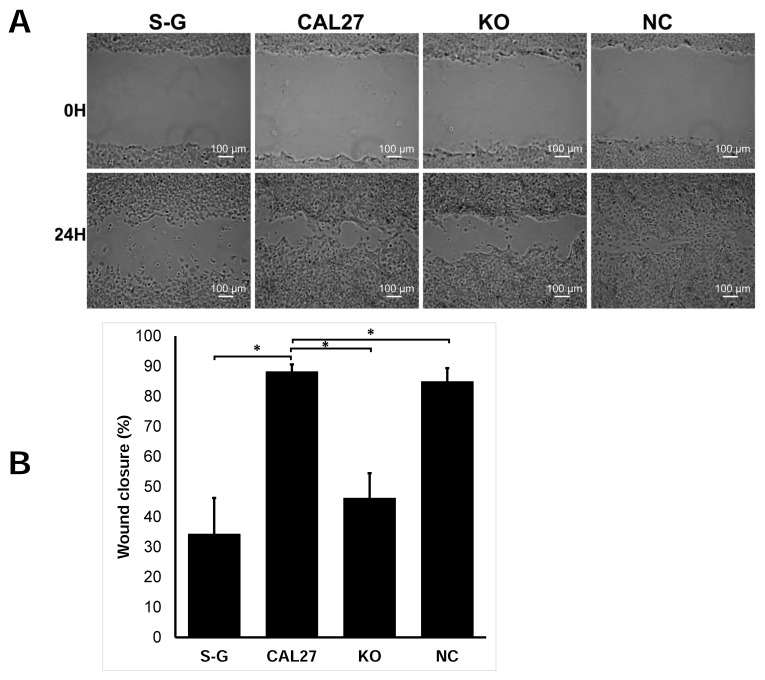
Knockout of CD82 in CAL 27 cells inhibited cell migration in wound healing assay. (**A**) Representative images of cell migration at the start (0 h) and 24 h. (**B**) Quantification of the data. The wounded areas were quantified using ImageJ. Cell migration is expressed as the percentage of wound closure. Wound closure (%) = (A0 − A24)/A0 × 100%, where A_0_ and A_24_ denote the wounded area at 0 and 24 h, respectively. The data were presented as means ± S.D. n ≥ 3, * *p* < 0.05.

In a wound healing assay, we observed that CAL 27 cells migrated faster than S-G cells, and the knockout of CD82 in CAL 27 inhibited cell migration (Figure 3A,B).

### 2.4. Transcriptomics and Proteomics Data Showed That CD82 Correlates with and May Regulate the Expression of S100 Family Members

**Figure 4:**
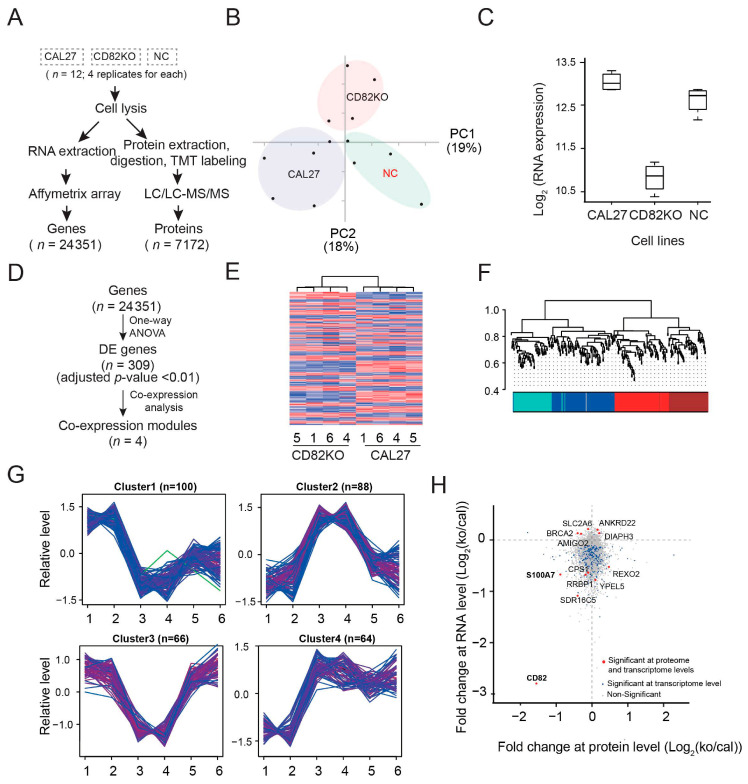
Knockout of CD82 downregulated S100A7 gene and protein expression in CAL 27 cells. The differentially expressed genes and proteins and co-expression network analysis of CAL 27 cells and CD82KO cells. (**A**) Flowchart showing transcriptomic and proteomic data processing. (**B**) Principal component analysis (PCA) plots of 12 samples (4 replicates per sample) using transcriptomic data. (**C**) Box plot showing CD82 mRNA level in CAL 27, CD82KO, and negative control cells (NC). (**D**) Diagram showing the identification of differentially expressed genes and co-expression modules. (**E**) Heat map of differentially expressed genes showing changes in expression between CD82KO cells and CAL 27 cells. Adjusted *p*-value < 0.01 was applied. (**F**) Four co-expression modules were identified from transcriptomic data. (**G**) Expression patterns of 4 co-expression modules. Each condition shows two points (CAL 27: 1,2; CD82KO: 3,4; negative controls (NC): 5,6). (**H**) Comparison of transcriptome and proteome data highlighting 11 differentially expressed genes/proteins at both mRNA and protein levels. S100A7, SDR16C5, RRBP1, and CPS1 were downregulated, whereas ANKRD22 and DIAPH3 were upregulated in CD82KO cells (adjusted *p*-value < 0.01). Among them, S100A7 was the most downregulated in CD82KO cells, suggesting that CD82 regulates S100A7 expression in CAL 27 cells.

The transcriptomics and proteomics analyses of 24,351 genes and 7172 proteins in CD82KO cells revealed alterations in over 300 genes and many proteins compared to CAL 27 cells. The differentially expressed genes and co-expression network analysis of CAL 27 cells and CD82KO cells are shown in Figure 4A–G. A comparison of the transcriptome and proteome data highlighted 11 differentially expressed genes/proteins at both the mRNA and protein levels. Notably, S100A7, SDR16C5, RRBP1, and CPS1 were downregulated, whereas ANKRD22 and DIAPH3 were upregulated in CD82KO cells (adjusted *p* value < 0.01). S100A7 was downregulated the most in CD82KO cells (Figure 4H), suggesting that CD82 may regulate S100A7 expression in CAL 27 cells.

### 2.5. Experiments Validated the Omics Results Show That CD82 Correlates with and May Regulate the Expression of S100 Family Members

#### 2.5.1. Real-Time PCR and Western Blotting Confirmed That CD82 Expression Correlates with and May Regulate S100 Proteins in CAL 27 and CD82KO Cells

**Figure 5:**
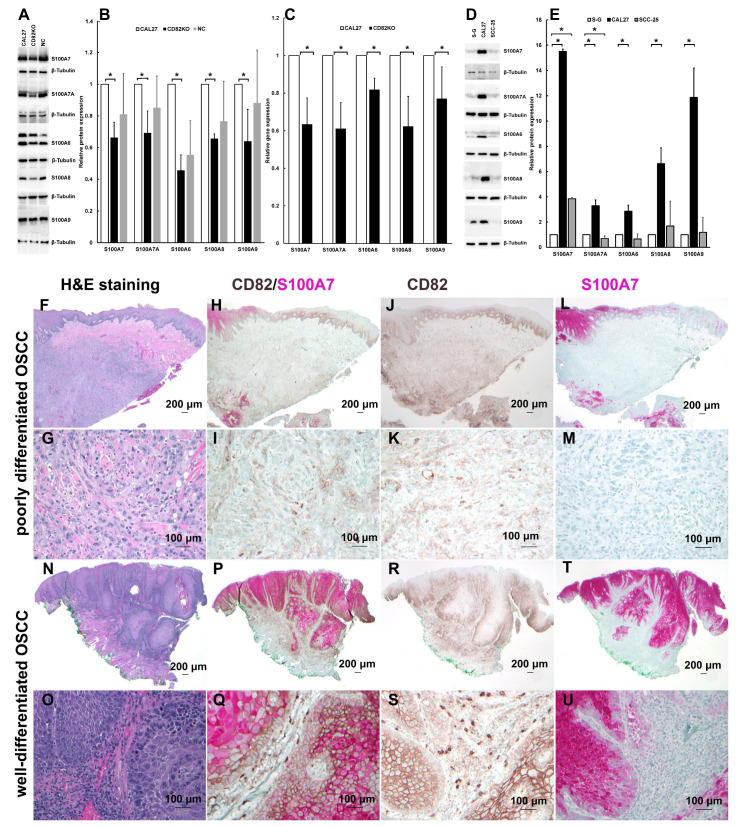
Experiments showed that CD82 correlates with and may regulate the expression of S100 family members. (**A**) Representative blots showing the expression of S100 family proteins in CAL 27, CD82KO, and negative control (NC) cells. β-tubulin as the loading control. (**B**) Relative protein expression levels quantified from Western blots using ImageJ. The expression levels of S100 family proteins were quantified relative to those in CAL 27 cells. * means statistically significant (*p* < 0.05). Replicates: n ≥ 3. (**C**) Relative gene expression levels were quantified from real-time PCR data; Tubulin (TUBB3) was used as the reference gene. * *p* < 0.05; n ≥ 9. (**D**) Representative blots showing the expressions of S100A7, S100A7A, S100A6, S100A8, and S100A9 in S-G, CAL 27, and SCC-25 cells. β-Tubulin was used as the loading control. (**E**) Relative protein expression levels quantified from Western blots using ImageJ. The expression levels of S100 family proteins were quantified relative to those in S-G cells. * *p* < 0.05; n ≥ 3. (**F**–**M**) Human tongue tissue with poorly differentiated OSCC. (**N**–**U**) Human tongue tissue with well-differentiated OSCC. (**F**,**H**,**J**,**L**,**N**,**P**,**R**, or **T**) Low-power image; rectangles mark the approximate area magnified in the high-power image below it. (**G**,**I**,**K**,**M**,**O**,**Q**,**S**, or **U**) High-power image. TS82b and MA1-91555 were used as the primary antibodies for CD82 and S100A7, respectively. The ultraView Universal (or Optiview) DAB Detection Kit was used to stain CD82, and ultraView Universal Alkaline Phosphatase Red Detection Kit was used for S100A7. Corresponding H&E staining sections were used as references.

The real-time PCR and Western blotting experiments confirmed that S100 family genes and proteins—namely S100A7, S100A7A, S100A6, S100A8, and S100A9—were significantly downregulated in CD82KO cells compared to CAL 27 cells (Figure 5A–C). That is, CD82 expression correlates with and may regulate S100A7, S100A7A, S100A6, S100A8, and S100A9 expressions.

#### 2.5.2. The Expression Levels of S100 Proteins Correlate to CD82 in CAL 27, SCC-25, and S-G Cells

Since CD82 is upregulated in CAL 27 cells but similar in SCC-25 cells when compared to S-G cells (Figure 1), we hypothesized that the expression levels of S100A7, S100A7A, S100A6, S100A8, and S100A9 are upregulated in CAL 27 cells and similar in SCC-25 cells when compared to S-G cells. Western blotting demonstrated that the expression levels of S100A7, S100A7A, S100A6, S100A8, and S100A9 are high in CAL 27 and comparable in SCC-25 when compared to S-G cells (D-E), which confirmed that S100 family proteins’ expression levels correlate to CD82 expression in CAL 27, SCC-25, and S-G cells (Figure 1 and Figure 5D,E).

#### 2.5.3. CD82 Correlates with and May Regulate S100A7 Expression in OSCC Tissue

We conducted CD82 and S100A7 single and dual staining immunohistochemistry (IHC) in FFPE human OSCC tissues using Hematoxylin and Eosin (H&E) staining as references.

In our limited study of two poorly differentiated FFPE human OSCC tissues by IHC and H&E, CD82 and S100A7 showed low or no expression in OSCC cells, similar to SCC-25 cells. The tissues are from males aged 40 and 57 years, subtype ‘SCC, conventional (keratinizing)’ according to the 5th WHO edition, with negative perineural and vascular invasion and incisional biopsies. This suggests that CD82 may regulate S100A7 expression in poorly differentiated OSCC tissue (Figure 5F–M).

Since many well-differentiated OSCC tissues have upregulated S100A7, we conducted IHC and H&E staining of five well-differentiated FFPE human OSCC tissues. The tissues are from males aged 54–84 years, subtype ‘SCC, conventional (keratinizing)’ according to the 5th WHO edition, with negative perineural and vascular invasion and incisional or excisional biopsies. We found that CD82 and S100A7 were highly expressed and overlapped at the parabasal cells, indicating that CD82 may regulate S100A7 expression. Meanwhile, in the tumoral islands, the highest expression of CD82 was found in less differentiated cells, while S100A7 was found in more differentiated cells.

These findings indicate that CD82 correlates with and may regulate S100A7 expression in OSCC tissues, but it may not be the only one that regulates S100A7 in OSCC tissues (Figure 5F–U).

### 2.6. Molecular Mechanisms of the Upregulation and Function of CD82 in CAL 27

#### 2.6.1. The 37–50 kDa CD82 Protein in CAL 27 Cells Is the Major CD82 Protein Molecule Upregulated in CAL 27 Compared to S-G Cells

**Figure 6:**
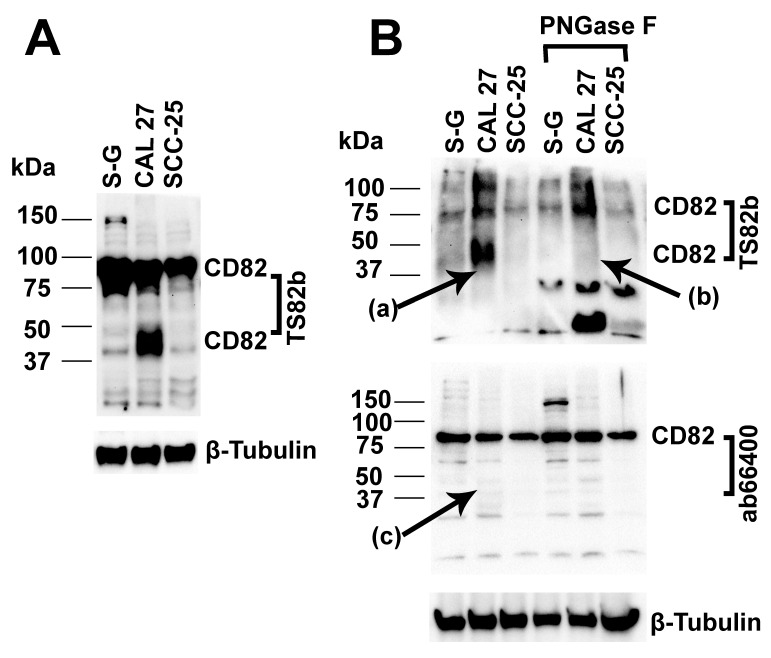
The 37–50 kDa CD82 protein in CAL 27 is upregulated, N-glycosylated and C-terminal truncated. (**A**) Under reducing conditions, Western blotting detects CD82 protein expression in S-G, CAL 27, and SCC-25 cells. (**B**) Deglycosylation of CD82 protein in S-G, CAL 27, and SCC-25 cells under reducing conditions. CD82 is N-glycosylated [arrowheads (a) and (b)]. CD82 is truncated at the C-terminus (lack of intact C-terminus) [arrowheads (a) and (c). Tubulin is used as a loading control.

We further explored the CD82 expression levels under reducing conditions in Western blotting. We found that, under reducing conditions, in addition to the 37–50 kDa band seen in the blot under non-reducing conditions, there is an additional band of CD82 detected at the molecular weight of 75 kD–100 kDa in S-G, CAL 27, and SCC-25 cells (Figure 6A). The 37–50 kDa band is the major CD82 band that is upregulated in CAL 27 when compared to S-G and SCC-25. That is, the 37–50 kDa molecular weight CD82 protein in CAL 27 cells may be responsible for the upregulation of CD82 and invasive metastasis in CAL 27.

#### 2.6.2. The 37–50 kDa CD82 Protein in CAL 27 Cells Is Glycosylated

N-glycosylation affects the CD82 protein’s molecular weight (42–100 kDa) and its interaction with integrins [19,20]. We hypothesized that the CD82 protein in CAL 27 might lack N-glycosylation and, therefore, cannot function in the same way as wild-type CD82. PNGase F is the most effective enzymatic method for removing almost all *N*-linked oligosaccharides from glycoproteins. Our glycoprotein deglycosylation experimental data showed that, if treated with PNGase F, the 37–50 kDa lower molecular weight CD82 band in CAL 27 diminished or degraded into small molecular weight proteins/peptides, which indicated that it was N-glycosylated [Figure 6, lane 2 and 5 arrowhead (a) and (b)]. Therefore, the 37–50 kDa CD82 in CAL 27 is N-glycosylated, rejecting our hypothesis.

#### 2.6.3. The 37–50 kDa CD82 Protein in CAL 27 Cells Is Truncated and Lacks an Intact C-Terminus

Next, we tested if the CD82 protein in CAL 27 is a wild type CD82 protein or if it is a truncated CD82 that lacks an intact C-terminus [21]. Our data demonstrated that although both TS82b and Abcam ab66400 anti-CD82 antibodies could detect the 75–100 kDa CD82 protein band, only the TS82b antibody could detect the 37–50 kDa CD82 protein band; the Abcam ab66400 anti-CD82 antibody was not able to detect the 37–50 kDa CD82 protein band (with or without PNGase F treatment). TS82b recognizes the large extracellular loop amino acids 17–82 and 84–242. Ab66400 is a synthetic peptide polyclonal antibody corresponding to human CD82 amino acid 250 to the C-terminus. This experiment indicates that the 37–50 kDa CD82 protein in CAL 27 is a truncated CD82 and lacks an intact C-terminus [Figure 6B arrowheads (a) and (c)]. This explains the upregulation of CD82, invasive metastasis, and poor outcome observed in the CAL 27 donor.

## 3. Discussion

S100A7 promotes cancer cell migration and contributes to the aggressive phenotype [22,23,24]. S100A7 is upregulated in breast, bladder, lung, head, and neck cancer tissues [25,26,27,28,29]. In the oral cavity, S100A7 is highly expressed in premalignant, well-differentiated OSCC tissues and accumulates in the nucleus of patients with head and neck SCC with poor prognoses. Analyzing tissue specimens from 93 patients with oral cavity cancer and 87 samples from normal oral mucosa found that S100A7 was significantly upregulated in cancerous samples [30]. However, the downregulation of S100A7 protein and its dual functionality in cancers were also reported [31,32,33]. Despite its high expression in the early stages of OSCC, S100A7 inhibits OSCC tumor growth, invasion, and progression. The downregulation of S100A7 in later stages promotes tumor growth and progression [33]. S100A7 exhibits heterogeneous and inducible characteristics in SCC. It acts as a dual regulator, promoting proliferation while suppressing differentiation [32]. The S100A7 protein is a promising biomarker for early-stage diagnosis and prognosis [27,30,34,35,36,37,38,39,40,41,42,43,44,45].

The overexpression of the brain-expressed X-linked 4 (BEX4) in CAL 27 cells increased the expressions of S100A7, S100A7A, S100A8, S100A9, and S100A12 [46]. Tumor suppressor genes CD82 and BEX4 likely regulate S100A7 expression.

In our study, CD82 knockout (KO) cell lines were generated through flow cytometry cell sorting to select the CD82 negative population. This method may result in a mixed population with varying degrees of CD82 expression, potentially influencing the specificity of our findings. However, the results may be more prominent if we have picked single-cell CD82 KO to establish the cell line.

Since S100 proteins promote cell migration [22,47,48,49], in CD82-upregulated CAL 27 cells, S100 family proteins are upregulated and promote cell migration. Knocking out CD82 downregulates S100 proteins and therefore inhibits cell migration. It is likely that CD82KO inhibits cell migration due to the downregulation of S100 proteins. CD82 likely regulates OSCC cell migration by regulating S100 protein expression. The 37–50 kDa CD82 protein in CAL 27 cells is the major CD82 protein molecule that is upregulated in CAL 27, and it is glycosylated and truncated. Further experiments like a rescue assay in CD82KO or overexpressing the truncated CD82 in SCC-25 or knockout S100A7 from CAL 27 or double-knockdown experiments are needed. These experiments would enable us to delineate whether the observed cell migration is predominantly driven by CD82, S100A7, or a synergistic effect of both.

Differential expressions of CD82 and S100 family proteins in CAL 27 and SCC-25 cells may represent two distinct subtypes of OSCC. Both cell lines were derived from poorly differentiated tongue squamous cell carcinoma tissues. However, SCC-25 was isolated from a 70-year-old male subject at T2N1; the CAL 27 cells were isolated from a 56-year-old OSCC male subject who died six months after his diagnosis, which implies that CAL 27 might be more invasive with a poorer prognosis than SCC-25 [50]. In addition, *Candida albicans* infection occurred during CAL 27 cell line establishment, and CD82 knockout mice were more susceptible to *C. albicans* than wild-type mice [50,51]. We speculate that the upregulation of CD82 and S100A7 in CAL 27 is part of the cells’ reaction to a fungal infection. Poorly differentiated OSCC with upregulated CD82 and S100A7 (S100 family proteins) might correlate with a more invasive phenotype and poor prognosis. Upregulated truncated CD82 and certain S100 proteins, such as S100A7, could serve as biomarkers for this subtype of poorly differentiated OSCC or SCC from other organ sites, but further investigation is necessary to validate if these findings can be generalized in SCC in general.

Most OSCC tissue specimens reported downregulated or negative CD82 expression; it takes more OSCC tissues to find poorly differentiated tissues with upregulated CD82 or S100A7 expression. However, studies have reported an upregulated expression of either CD82 or S100A7 in OSCC tissues with larger sample sizes, suggesting possible concurrent upregulation. Additional studies using primary cells and more OSCC tissues are needed to validate these findings.

How is CD82 expression regulated in normal cells and upregulated in poorly differentiated OSCC tissue subtype? Does this occur through transcriptional regulation or post-translational modification of the CD82 protein? How does CD82 function in this upregulated oral cancer cell subtype to regulate their progression and metastasis?

Possible mechanisms for the loss of CD82 gene function are the loss of heterozygosity or allelic imbalances at the CD82 locus on human chromosome 11p11.2, faster-than-normal ubiquitin degradation, altered transcriptional regulation, the production of a splice variant, a dominant-negative protein, and post-translational modification of the CD82 protein [3,52,53,54].

Our glycoprotein deglycosylation using PNGase F treatment experiment demonstrated that the 37–50 kDa CD82 in CAL 27 is N-glycosylated. Therefore, post-translational modification, such as a lack of glycosylation, does not seem to be the mechanism that prevents CD82 from suppressing metastasis in CAL 27.

KAI1 is another name for CD82. Truncated KAI1 or alternatively spliced (AS) KAI1 is associated with invasive metastasis and poor patient outcomes—except when (AS) KAI1 is expressed at low levels in human bladder cancers [1,21,55]. Unlike the wild-type CD82, the truncated KAI1 can be detected by the TS82b antibody but not the Abcam EPR4112 antibody. The TS82b antibody targets the extracellular loop, while the EPR4112 antibody targets the C-terminal intracellular domain of the protein. That is, the truncated KAI1 lacks an intact C-terminus [21]. We used an ab66400 anti-CD82 antibody that is similar to the Abcam EPR4112 antibody but with a known epitope sequence corresponding to human CD82 amino acid 250 to the C-terminus. We found that the 37–50 kDa CD82 protein in CAL 27 is a truncated CD82 and lacks an intact C-terminus. This truncated CD82 expression explains the invasive metastasis and poor patient outcome of CAL 27 donor.

Further investigations are required to determine whether this mechanism is generalized in SCC from other organ sites.

## 4. Materials and Methods

### 4.1. Cell Lines

CAL 27 and SCC-25 human tongue squamous cell carcinoma cell lines were obtained from American Type Culture Collection (ATCC, Manassa, VA, USA). S-G cell line was obtained from F. H. Kasten, East Tennessee State University, Quillen College of Medicine, Johnson City, TN [56,57]. S-G, CAL 27, and SCC-25 cells were cultured in DMEM or DMEM/F-12 medium at 37 °C supplied with 5% CO_2_. Both media contained 10% (*v*/*v*) fetal bovine serum and penicillin-streptomycin (1%). All reagents and supplies were purchased from Thermo Fisher Scientific (Thermo Fisher Scientific, Waltham, MA, USA) unless otherwise specified.

Antibodies and key resources are listed in the Supplementary Materials Table S1.

### 4.2. Immunoblotting

Immunoblotting was conducted as described [58]. Briefly, cells (2 × 10^6^) were lysed in a radioimmunoprecipitation assay (RIPA) buffer containing Tris-buffered saline (TBS), 1% Triton X-100, 1× EDTA-free complete protease cocktail inhibitor (Roche, Indianapolis, IN, USA), 2 mM sodium orthovanadate, 2 mM β-glycerophosphate, 1 mM phenylmethylsulfonyl fluoride (PMSF), and 2 mM sodium fluoride. The lysates were sonicated on ice and then centrifuged at 14,000 rpm at 4 °C for 10 min. The supernatants (total protein) were measured using BCA protein assay. The samples were separated by SDS-PAGE under reducing (Figure 6) or nonreducing conditions, transferred onto nitrocellulose membrane, blocked with 5% milk in TBS at room temperature for 1 h, incubated with primary antibody at 4 °C overnight, washed with TBST, and then incubated with HRP conjugated anti-rabbit or anti-mouse secondary antibody. Finally, proteins were visualized using an enhanced ECL reagent (GE Healthcare Systems, Chicago, IL, USA) under a ChemiDoc Imager (BioRad, Hercules, CA, USA) and quantified using ImageJ 1.52a/Java 1.8.0_112 software (NIH, Bethesda, MD, USA).

### 4.3. Immunofluorescence Microscopy

Cells were cultured on glass coverslips overnight, fixed with 4% paraformaldehyde (Santa Cruz Biotechnology, Santa Cruz, CA, USA) at room temperature for 20 min, permeabilized with 0.5% Triton X-100 for 15 min, and washed with PBS. After blocking with 1% BSA in TBS at room temperature for 1 h, cells were incubated with the CD82 primary antibody at 4 °C overnight, washed with PBS, and then incubated with fluorescein (FITC) conjugated secondary antibodies (Sigma-aldrich, St. Louis, MO, USA) for 1 hour. Finally, samples were mounted with medium containing 4’,6-diamidino-2-phenylindole (DAPI) to visualize nuclei and imaged using a Zeiss Axio Observer Z1 microscope equipped with AxioCam MRM camera (Carl Zeiss Microscopy, White Plains, NY, USA).

### 4.4. Generate CD82 Knockout CAL 27 Cells Using CRISPR-Cas9

To knock out CD82 gene in CAL 27 cells, we designed two TrueGuide Synthetic guide RNA sequences, CD82CHOP1sgRNA and CD82sgRNA, using the CHOPCHOP web tool and Invitrogen TrueDesign Genome Editor [59,60]. CD82CHOP1sgRNA: Target sequence GCCCATGTTGAAGTAGAAGAGGG. Target locus Chr.11: 44615404 on GRCh38. Strand −. Targeted isoform(s): NM_002231, isoform 1. CD82sgRNA: Target sequence: AGACTACAACAGCAGTCGCGAGG. Target locus Chr.11: 44615313 on GRCh38. Strand +. Targeted isoforms: NM_001024844.1, NM_002231.3, XM_006718223.2, and XM_011520067.2. The TrueGuide™ human negative control—sgRNA non-targeting 1—was used as control. Invitrogen synthesized the CRISPR guide RNAs.

We transfected sgRNAs into CAL 27 cells using a CRISPR-Cas9 kit. Briefly, cells were seeded in 6-well plates (2 × 10^6^ cells/well) and transfected with sgRNA/Cas9v2 nuclease complex using Lipofectamine CRISPRMAX^TM^ reagent for 2–3 days in Opti-MEM^TM^ 1 medium. After removing the medium, cells were washed with PBS, trypsinized, and subcultured for cell sorting.

### 4.5. CD82 Immunofluorescence Staining and Flow-Activated Cell Sorter (FACS)

Wild-type CAL 27, CD82 sgRNAs, and negative control transfected cells were detached and immunostained with an anti-CD82 primary antibody (TS82b) and FITC-conjugated anti-mouse IgG secondary antibody for FACS analysis. The CD82 negative population in knockout cells was sorted and subcultured as knockout cell lines.

### 4.6. Wound Healing Assay

Cells were plated into a six-well plate (2 × 10^6^ cells/well) and cultured in complete medium overnight. Cell monolayers were scratched with 200 μL pipette tips, washed three times with 1× PBS, and incubated for 24 h. Wounds at the start and at 24 h were imaged under a PrimoVert inverted microscope with a camera (Zeiss) and quantified using ImageJ. The migration rate is the percentage of wound closure.

### 4.7. Microarray Transcriptomics in CAL 27 and CD82KO Cells

CAL 27 and CD82KO cells were cultured in 100 mm culture dishes for 48 h. mRNAs were extracted and purified using Qiacube and RNeasy Mini QIAcube Kit (QIAGEN Inc., Valencia, CA, USA). The quality and quantity of RNAs were tested using NanoDrop™ OneC Microvolume UV-Vis Spectrophotometer and Agilent Bioanalyzer (Agilent Technologies, Santa Clara, CA, USA).

Gene expression was profiled using Affymetrix GeneChip Probe Arrays (Affymetrix Clariom™ S Array, human). Two hundred nanograms of DNased total RNA was amplified, labeled, and fragmented using Affymetrix WT amplification kit. Samples were hybridized overnight, washed, stained on Affymetrix fluidics 450 station, and then scanned using GCS3000.

#### Data Analysis

Data were normalized using quantile normalization (i.e., Robust Multichip Average (RMA)). Batch effects were corrected using “Remove Batch Effect” function in the LIMMA 3.43.5 software. Differentially expressed genes were detected using analysis of variance (ANOVA). Benjamini–Hochberg (BH) method was used for multiple comparison correction with an adjusted *p*-value of 0.01. Co-expression network was constructed using the WGCNA package in R [61]. A weighted adjacency matrix was calculated based on the β parameter to meet the scale-free topology criterion. Gene functional enrichment was analyzed using Fisher’s Exact Test with a significance threshold of a *p*-value of <0.01.

### 4.8. Proteomics and Proteins Profiling

#### 4.8.1. Sample Processing

Samples were processed as described [62]. Briefly, protein samples from CAL 27, CD82KO, and negative control cell lysates were centrifuged and supernatants were collected; protein concentration was measured using BCA assay. Each biological replicate (60 g of protein in 60 µL of lysis buffer) underwent a Mass Spec Sample Prep Kit for Cultured Cells (Pierce™). The samples were reduced with 10 mM DTT (45 min, 50 °C), followed by 50 mM iodoacetamide alkylation (20 min, room temperature, avoid light); subsequently, proteins were precipitated and washed with freezer-cold (−20 °C) acetone (five volumes, overnight; 50 µL, 90%), air-dried, and re-dissolved in a digestion buffer (100 mM TEAB, pH 8.3, 100 µL). Proteins were digested using Lys-C enzyme (1:100 ratio, 2 h at 37 °C) and trypsin (1:50 ratio, overnight). Peptide concentrations were measured using Quantitative Colorimetric Peptide Assay (Pierce).

Samples were labeled with TMT Mass Tag (20 µg of peptides per sample).

The labeled peptide mixture was vacuum-dried, reconstituted (90 µg in 300 µL) and fractionated using High pH Reversed-Phase Peptide Fractionation kit (Pierce), and eight fractions were collected and vacuum-dried.

#### 4.8.2. LC-MS/MS

The dried peptide fraction (~11 µg) was re-dissolved in a loading buffer (3% acetonitrile, 0.05% TFA, in 100 µL), and an aliquot (0.55 µg in 5 µL) was analyzed using an Orbitrap Fusion Lumos mass spectrometer with Ultimate 3000RSLCnano UHPLS system (LC-MS/MS). The peptides were first trapped and then separated on Acclaim PepMap columns (100 nanoViper column, 75 µm × 20 mm, 5 µL/min flow rate; then, an RSLC nanoViper column, 75 µm × 500 mm, C-18, 2 µm, 100 Å, 300 nl/min flow rate, water and acetonitrile with 0.1% formic acid as solvents A and B). MS3 Synchronous Precursor Selection (SPS) acquisition method with the following parameters were used: MS1 scan, Orbitrap analyzer—a resolution of 120,000 (FWHM, *m*/*z* = 200), MS2 scan, Ion Trap—charge state of 2–6, intensity minimum of 5000, isolation window of 0.7 *m*/*z*, and fragmented (CID, 35% NCE). Peptide ions were isolated (2 *m*/*z* window), the top 10 MS2 fragment ions underwent further fragmentation (HCD, 65% NCE), and reporter ions intensities were determined in the Orbitrap analyzer at the resolution of 50,000 (FWHM, *m*/*z* = 200).

#### 4.8.3. Post-Acquisition Analysis of Raw MS Data

JUMP and JUMPq programs were used for database search and protein quantification [63,64,65]. The concatenated target decoy strategy was used to filter the PSMs to achieve ~1% FDR of peptide identification [66,67]. Batch effect was controlled using a pooled control sample and the “Remove Batch Effect” function in LIMMA 3.43.5 software employing linear correction. Group comparison was conducted using analysis of variance (ANOVA) with the Benjamini–Hochberg (BH) method for multiple comparison correction, using an adjusted *p*-value of 0.01 to detect differentially expressed proteins. Protein co-expression networks were constructed using the same approach as for microarray data.

### 4.9. Primers and Probes

Real-time PCR primers and probes were designed using the ProbeFinder version 2.53 Assay Design Software (Roche, Indianapolis, IN, USA). The primer oligos were purchased from the Integrated DNA Technologies company (Integrated DNA Technologies, Coralville, IA, USA). Universal Probe Library (UPL) probes were purchased from Roche. The relative changes in gene expression were analyzed using the 2^−ΔΔCT^ method [68].

### 4.10. CD82 and S100A7 Single and Dual Staining IHC in FFPE Human OSCC Tissue

CD82 and S100A7 single and dual staining and H&E staining were performed on a BenchMark ULTRA IHC/ISH System (Roche, Indianapolis, IN, USA). The paraffin-embedded tissue sections (4 μm) were de-paraffinized and incubated with CD82 and S100A7 antibodies (TS82b and MA1-91555), followed by Ventana Medical Systems ultraView Universal (or Optiview) DAB (3,3′-diaminobenzidine tetrahydrochloride) Detection Kit for CD82 and ultraView Universal Alkaline Phosphatase Red Detection Kit (Roche Indianapolis, IN, USA) for S100A7. The slides were then counterstained with hematoxylin, mounted, and imaged using an Olympus BX51 microscope with an InfinityX camera and the Infinity analyze 3 6.5.5 software (Lumenera Corporation, Ottawa, ON, USA).

### 4.11. Glycoprotein Deglycosylation and Truncated CD82 Detection

Glycoprotein deglycosylation was carried out using PNGase F kit following the manufacturer’s protocol. An amount of 20 µg of cell lysates of SG, CAL-27, or SCC-25 were treated with or without PNGase F (1–2 µL) in the glycoprotein denaturing buffer and denatured at 100·°C for 10 min. Denatured samples were added to 2 μL of 10× G7 Reaction Buffer and 2 μL of 10% NP40 and adjusted to a volume of 20 µL. Samples were then boiled at 37 °C for 1 h, separated by SDS-PAGE under reducing conditions, and transferred onto a nitrocellulose membrane. Finally, samples were probed with TS82b or ab66400 anti-CD82 antibodies and respective secondary antibodies to detect the glycosylation of CD82.

Truncated CD82 or the alternative splicing form CD82 was detected simultaneously with the deglycosylation assay using two anti-CD82 antibodies. Truncated CD82 lack of intact C-terminus can only be detected by TS82b but not ab66400.

### 4.12. Statistical Analysis

Student’s t-test or one-way analysis of variance (ANOVA) using Excel and SigmaPlot version 12.5. *p* < 0.05 was considered statistically significant. The data represent mean ± standard deviation (SD) for three independent experiments.

## 5. Conclusions

CD82 expression varies in OSCC cell lines; it is upregulated in CAL 27 and comparable in SCC-25 cells. The knockout of CD82 in CAL 27 cells downregulated S100 proteins and inhibited cell migration. CD82 correlates with and may regulate the expression of S100 family proteins, including S100A7, S100A7A, S100A6, S100A8, and S100A9 in OSCC cell lines and tissue. This association between CD82 and S100 family proteins in poorly differentiated OSCC could be a novel mechanism through which CD82 regulates migration and metastasis. The 37–50 kDa CD82 protein in CAL 27 cells is upregulated, glycosylated, and truncated. This truncated CD82 expression may explain the invasive metastasis and poor outcome of the CAL 27 donor. The combination of upregulated truncated CD82 and upregulated S100A7 could be a potential biomarker for a distinct subtype of poorly differentiated OSCC or SCC with poor prognosis. Differing alternative splicing from wild-type CD82 may elucidate the contradictory functions and pave the way for CD82 as a prognostic biomarker and therapeutic target.

## Data Availability

The data are contained within the article or supplementary material.

## Supplementary Materials

The following supporting information can be downloaded at https://www.mdpi.com/article/10.3390/ijms25052659/s1.
