# Supplementary material for: Tetraspanin CD82 Correlates with and May Regulate S100A7 Expression in Oral Cancer
Source: Int J Mol Sci. 2024 Feb 24;25(5):2659. doi: 10.3390/ijms25052659 (PMC10932236; doi:10.3390/ijms25052659)
Supplement: Supplementary file 1 [file ijms-25-02659-s001.zip › ijms-2859011-supplementary.pdf]

Article

Title: Tetraspanin CD82 Correlates with and may Regulate S100A7 Expression in Oral Cancer

Kiran Kumar Reddi [1], Weiqiang Zhang [2,3,4], Shokoufeh Shahrabi-Farahani [5], Kenneth Mark Anderson [5], Mingyue Liu [1], David Kakhniashvili [6], Xusheng Wang [7*✉], and Yanhui H. Zhang [1*✉]

1. Supplemental Table S1: Key Resources

REAGENT or RESOURCE	SOURCE	IDENTIFIER
Antibodies		
Mouse monoclonal anti-Human CD82 (Clone TS82b)	Cell sciences	Cat# CDM457 Cat#857.790.000, RRID: AB_2076514
Rabbit polyclonal anti-Human CD82	Abcam	Cat# ab66400; RRID: AB_1640343
Mouse monoclonal anti β-Tubulin (Clone TUB 2.1)	Sigma-Aldrich	Cat# T4026
FITC goat anti-rabbit IgG	Sigma-Aldrich	Cat# F0382
FITC goat anti-mouse IgG	Sigma-Aldrich	Cat# F2012
Goat anti-rabbit IgG (H+L) Secondary Antibody, HRP	Thermo Fisher Scientific	Cat# 65-6120 RRID: AB_2533967
Goat anti-mouse IgG (H+L) Secondary Antibody, HRP	Thermo Fisher Scientific	Cat# 31430, RRID: AB_228307
Mouse monoclonal anti-Human S100A7 (47C1068)	Thermo Fisher Scientific	Cat# MA1-91555; RRID: AB_2183976
Rabbit polyclonal anti-Human S100A6	Thermo Fisher Scientific	Cat# PA5-31862; RRID: AB_2549335

Rabbit polyclonal anti-Human S100A8	Thermo Fisher Scientific	Cat# PA5-86063; RRID: AB_2802864
Rabbit polyclonal anti-Human S100A7A	Aviva Systems Biology	Cat# OACD01125; RRID:
Rabbit polyclonal anti-Human S100A9	Thermo Fisher Scientific	Cat# PA1-46489; RRID: AB_10977234
Normal Mouse IgG control	Santa Cruz	Cat# sc-2025; RRID: AB_ 737182
Normal Rabbit IgG control	Cell Signaling Technology	Cat# 2729S; RRID: AB_ 1031062
Chemicals, Peptides, and Recombinant Proteins		
Lipofectamine™ CRISPRMAX™ Cas9 Transfection Reagent	Invitrogen / Thermo Fisher Scientific	Cat# CMAX00008
TrueCut™ Cas9 Protein v2	Invitrogen / Thermo Fisher Scientific	Cat# A36498
Prolonged gold anti-fade reagent with 4,6-diamidino-2-phenylindole (DAPI)	Invitrogen / Thermo Fisher Scientific	Cat# P36931
Western Lightning Plus-ECL Chemiluminescence reagent	Perkin Elmer	Cat# NEL105001EA
Micro BCA Protein Assay Kit	Thermo Fisher Scientific	Cat# 23235
PMSF	Sigma-Aldrich	Cat# 30976261
Triton X-100	Sigma-Aldrich	Cat# T8787
β-Glycerophosphate	Sigma-Aldrich	Cat# 9422
Sodium fluoride	Sigma-Aldrich	Cat# S7920

Sodium orthovanadate	Sigma-Aldrich	Cat# 450243
Tris-EDTA buffer pH 8	Invitrogen / Thermo Fisher Scientific	Cat# AM9849
1M Tris pH 8	Invitrogen / Thermo Fisher Scientific	Cat# AM9856
Pierce™ Mass Spec Sample Prep Kit for Cultured Cells	Thermo Fisher Scientific	Cat# 84840
Thermo Scientific™ Pierce™ Quantitative Peptide Assays & Standards	Thermo Fisher Scientific	Cat# 23275
TMT Mass Tag Labeling reagent kit	Thermo Fisher Scientific	Cat# A34808
Pierce High pH Reversed-Phase Peptide Fractionation kit	Thermo Fisher Scientific	Cat# 84868
4% Paraformaldehyde	Santa Cruz	Cat# sc-281692
1M Tris/Glycine/SDS buffer	Bio-Rad	Cat# 161-0772
1M Tris/Glycine buffer	Bio-Rad	Cat# 161-0771
Laemmli Sample Buffer	Bio-Rad	Cat# 161-0747
Precision Plus Protein Dual Color Standards	Bio-Rad	Cat# 161-0374
Tween-20	Bio-Rad	Cat# 170-6531
Sodium deoxycholate	Sigma-Aldrich	Cat# D6750
Non-Fat Dry Milk Blotting Grade Blocker	Bio-Rad	Cat# 170-6404
EDTA-free complete protease cocktail inhibitor	Roche Molecular Systems	Cat# 04693132001
DMEM (1X) Liquid High Glucose, Pyruvate	Invitrogen/ Thermo Fisher Scientific	Cat# 11995-065
DMEM/F-12, HEPES	Invitrogen / Thermo Fisher Scientific	Cat# 11330-032

Fetal Bovine Serum	Invitrogen / Thermo Fisher Scientific	Cat# 10437-028
Goat Serum	Invitrogen / Thermo Fisher Scientific	Cat# 16210
Penicillin-Streptomycin	Invitrogen / Thermo Fisher Scientific	Cat# 15140-122
0.25% Trypsin EDTA	Invitrogen / Thermo Fisher Scientific	Cat# 25200-056
DPBS	Gibco/ Thermo Fisher Scientific	Cat# 10010-023
Protein G sepharose 4 fast flow	GE-Healthcare	Cat# GE17-0618-01
Protein A sepharose CL-4B	GE-Healthcare	Cat# GE17-0780-01
Critical Commercial Assays		
ultraView Universal DAB Detection Kit	Roche Diagnostics GmbH	Cat# 760-500, RRID:AB_2753116
ultraView Universal Alkaline Phosphatase Red Detection Kit	Roche Diagnostics GmbH	Cat# 760-501
Deposited Data		
Experimental Models: Cell Lines		
Smulow-Glickman (S-G) human gingival epithelial cell line	F.H. Kasten	[56,57]
CAL 27 (Human squamous cell carcinoma, Epithelial)	ATCC	Cat# CRL-2095™ RRID:CVCL_1107
SCC-25 (Human Squamous cell carcinoma, Epithelial)	ATCC	Cat# CRL-1628™ RRID:CVCL_1682
Oligonucleotides		

CD82CHOP1 sgRNA (TrueGuide synthetic human CD82 sgRNA)<br>Target:GCCCATGTTGAAGTAGAAGAGGG<br>sgRNA: GCCCAUGUUGAAGUAGAAGA<br>Target locus Chr.11: 44615404 on GRCh38, Strand -,<br>Targeted Isoform(s): NM_002231, isoform 1.	Invitrogen / Thermo Fisher Scientific	Cat# A35513
CD82 sgRNA<br>(TrueGuide™ synthetic human CD82 sgRNA)<br>Target: AGACTACAACAGCAGTCGCGAGG<br>sgRNA: AGACUACAACAGCAGUCGCGAGG<br>Target locus Chr.11: 44615313 on GRCh38, Strand +,<br>Targeted Isoforms: NM_001024844.1, NM_002231.3, XM_006718223.2, XM_011520067.2. A	Invitrogen / Thermo Fisher Scientific	Cat# A35510
TrueGuide™ human negative control, sgRNA non-targeting 1	Invitrogen / Thermo Fisher Scientific	Cat# A35526
CD82L gaaagcagaacccgcaga<br>CD82R ccagtgcagctggtcaca<br>Probe; 29	Integrated DNA technologies; Roche Molecular Systems	
S100A7L ccaaacacacacatctcactca<br>S100A7R tcagcttgagtgttgctcatc<br>Probe; 33	Integrated DNA technologies; Roche Molecular Systems	
S100A7AL tctcactcatccttctactcgtga<br>S100A7AR tcagcttgagtgttgctcatc<br>Probe; 48	Integrated DNA technologies; Roche Molecular Systems	
S100A6L actgcgacacagcccatc<br>S100A6R gaagatggccacgaggag<br>Probe; 23	Integrated DNA technologies; Roche Molecular Systems	
S100A8L gccaagcctaaccgctataa<br>S100A8R atgatgcccacggacttg<br>Probe; 17	Integrated DNA technologies; Roche Molecular Systems	
S100A9L gtgcgaaaagatctgcaaaa<br>S100A9R tcagctgcttgtctgcattt<br>Probe; 85	Integrated DNA technologies; Roche Molecular Systems	

TUBB3L gcacacagaccggacaca<br>TUBB3R gaggcctcgttgtagtagacg<br>Probe 78	Integrated DNA technologies; Roche Molecular Systems	
Software and Algorithms		
CHOPCHOP web tool	chopchop.rc.fas.harvard.edu; https://chopchop.cbu.uib.no/	[59,60]
Excel 2016	Microsoft	https://www.office.com/
ImageJ software	https://imagej.nih.gov/ij/	
Infinity analyze 3 6.5.5 software	Lumenera corporation, Ottawa, ON	https://www.lumenera.com/infinity-analyze-and-capture-for-windows.html
Proteome Discoverer 2.2	Thermo Fisher Scientific	https://www.thermofisher.com/
JUMP program	https://github.com/JUMPSuite/JUMP	[65]
ProbeFinder Assay Design Software	Roche Molecular Systems	https://lifescience.roche.com/en_us/articles/Universal-ProbeLibrary-System-Assay-Design.html
SigmaPlot 12.5 software	Systat Software, Inc	https://systatsoftware.com/
TrueDesign Genome Editor	Thermo Fisher Scientific website	https://www.thermofisher.com/us/en/home/life-science/genome-editing/geneart-crispr/geneart-crispr-search-and-design-tool.html.
Zen Zeiss 2012 SP2 blue microscope software	https://www.zeiss.com/corporate/us/home.html	https://www.zeiss.com/microscopy/us/products/microscope-software/zen-lite.html
